# miRNA in Ischemic Heart Disease and Its Potential as Biomarkers: A Comprehensive Review

**DOI:** 10.3390/ijms23169001

**Published:** 2022-08-12

**Authors:** Amanda Shen-Yee Kong, Kok-Song Lai, Swee-Hua Erin Lim, Sivakumar Sivalingam, Jiun-Yan Loh, Sathiya Maran

**Affiliations:** 1 School of Pharmacy, Monash University Malaysia, Jalan Lagoon Selatan, Bandar Sunway, Subang Jaya 47500, Malaysia; 2 Health Sciences Division, Abu Dhabi Women’s College, Higher Colleges of Technology, Abu Dhabi 41012, United Arab Emirates; 3 Department of Cardiacvascular and Thoracic Surgery, National Heart Institute, 145, Jalan Tun Razak, Kuala Lumpur 50400, Malaysia; 4 Centre of Research for Advanced Aquaculture (CORAA), UCSI University, Cheras, Kuala Lumpur 56000, Malaysia

**Keywords:** microRNAs, ischemic heart disease, therapeutic targets, diagnostics, cardiac biomarkers

## Abstract

Ischemic heart disease (IHD) constitutes the leading global cause of mortality and morbidity. Although significant progress has been achieved in the diagnosis, treatment, and prognosis of IHD, more robust diagnostic biomarkers and therapeutic interventions are still needed to circumvent the increasing incidence of IHD. MicroRNAs (miRNAs) are critical regulators of cardiovascular function and are involved in various facets of cardiovascular biology. While the knowledge of the role of miRNAs in IHD as diagnostic biomarkers has improved, research emphasis on how miRNAs can be effectively used for diagnosis and prognosis of IHD is crucial. This review provides an overview of the biology, therapeutic and diagnostic potential, as well as the caveats of using miRNAs in IHD based on existing research.

## 1. Introduction

Ischemic heart disease (IHD) remains a burden to individuals and health systems worldwide, accounting for 49.2% of total cardiovascular deaths in 2019 [1]. The global disease burden is 126.5 million, and in the United States, someone dies from coronary disease every 36 s [2,3]. Studies suggest that the incidence and prevalence rates of IHD increase with age, and the age distribution indicates clearly that 70% of those 26 to 39 were diagnosed with coronary atherosclerosis sub-clinically [4]. The modifiable contributing factors for IHD are hypertension, diabetes mellitus (DM), lipid imbalance, and cigarette smoking [5]. These can be classified under metabolic syndrome, which activates plaque formation, promotes thrombosis and inflammation, and increases the risk for cardiovascular disease development [6]. A range of debilitating conditions such as collagen deposition and elastin reduction regularly exist in older people, resulting in a poor prognosis of IHD with disabilities and impairment in quality of life, worsening their condition [7,8].

The current diagnosis of IHD relies on coronary angiography to perform the anatomical and functional assessment of the coronary artery [9]. However, there remains the possibility for misdiagnosis of the disease due to technical factors, the complexity of coronary anatomy, and plaque configuration [10]. The use of local anaesthesia and contrast material may also pose a risk of health-related complications to patients.

Non-imaging biomarkers used for IHD diagnosis are cardiac troponin (cTn)—both I and T forms (cTnl and cTnT) and high-sensitivity C-reactive protein [11]. Despite having the advantages of being non-invasive and without radiation exposure compared to coronary angiography, several disadvantages of these were discussed by Meder and colleagues [12]. Namely, reduced sensitivity, not sufficient specificity or they do not allow timely diagnosis. Hence, the discovery of novel microRNA (miRNA) techniques offers high prospects for their potential to detect IHD in its early stages [13].

**Figure 1:**
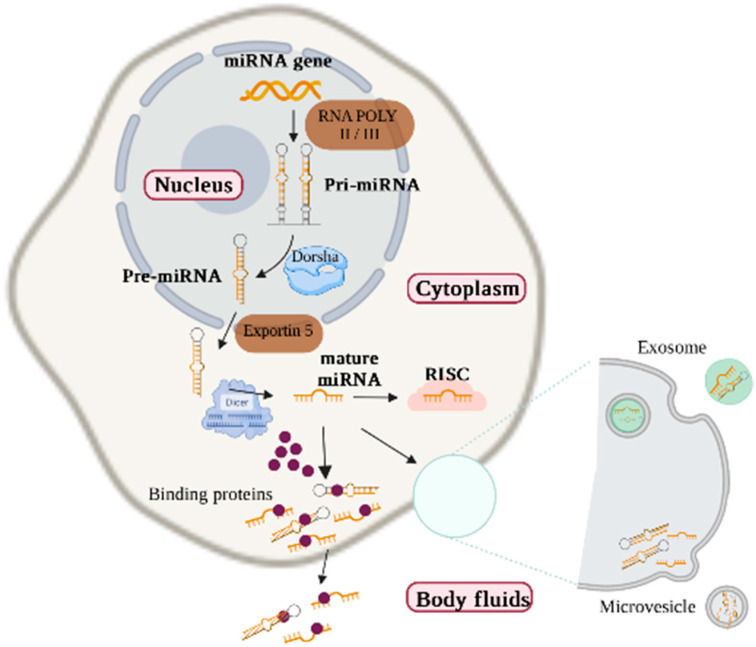
The biogenesis of miRNA and how they are being transported to various body fluids.

The miRNAs are intercellular endogenous RNA molecules of approximately 22 non-coding nucleotides that regulate the expression of targeted genes by mRNA degradation and translational repression [14]. Interestingly, more than one miRNA can target and bind to the same 3′UTR site of mRNA, suggesting its role in almost every biological process [15]. Circulating miRNAs travel in the blood and other bodily fluids such as saliva, urine, breast milk, and semen, and thus are postulated as a potential diagnostic and prognostic indicators for heart failure [16,17,18]. Researchers in this regard compare the expression level between patients and healthy controls to investigate their specific role as molecular biomarkers for IHD [19]. Circulating miRNAs are protected from the endogenous ribonuclease (RNases) activity by transport particles such as exosomes, microvesicles, or through binding to a protein complex or high-density lipoproteins (HDL) [20,21] (Figure 1). Mainly, extracellular vesicle-carried miRNAs have been reported as significantly expressed in IHD-DM patients and have excellent diagnostic efficacy [22].

Circulating miRNAs fulfil most of the ideal biomarker requirements that include high stability, specifically tissue-derived, and are easily detected by sequence-specific amplification [13]. The miRNAs were also reported to act by either destroying the target mRNA lowering its seric concentration or blocking the translation [23]. Thus, circulating miRNAs have been considered promising biomarkers for rapid diagnosis and disease management of IHD. This review aims to critically appraise and discuss the potential role of miRNAs in IHD and their use as potential biomarkers.

## 2. Current Outlooks for miRNAs in Ischemic Heart Disease

**Figure 2:**
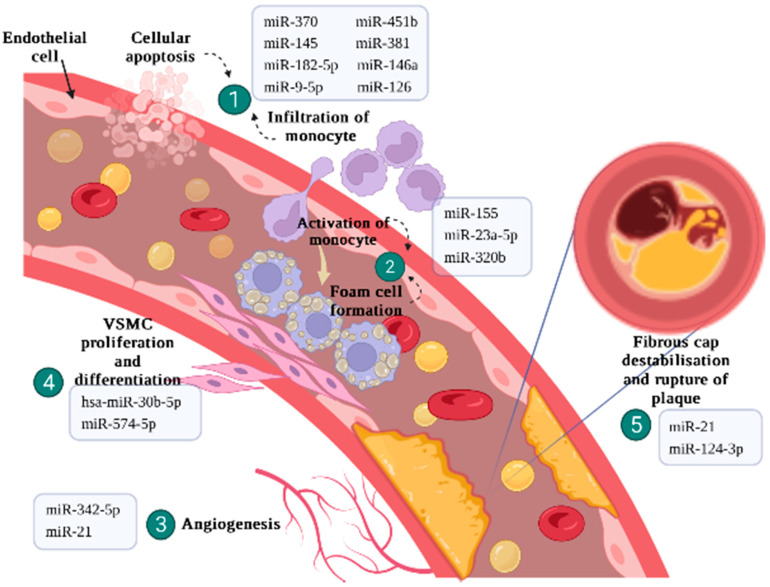
Involvement of miRNAs in the different steps of atherosclerosis. Plaque formation begins with endothelial dysfunction, where the regulating of endothelial cells is imbalanced and initiates the infiltration of leukocytes and monocytes into coronary blood vessels [27]. Upon activation, macrophages will transform into foam cells and trigger plaque angiogenesis to compensate for the ischemic condition. The VSMCs will continue to migrate and proliferate, subsequently, fibrous cap destabilisation and rupture of plaque, leading to the final stage of thrombosis contributing to acute coronary syndrome (ACS) [28].

Atherosclerosis plays a crucial role in the pathophysiology of IHD [9]. Hypercholesterolemia increases the chance of lipoprotein oxidation and macrophages activation [24]. These further initiate the proliferation of vascular smooth muscle cells (VSMCs) to produce fibrous caps that contribute to plaque stability [25]. Atherosclerotic lesions that grow slowly over time can trigger the formation of thrombosis and the inflammatory response which releases pro-inflammatory molecules to destabilise the plaque. This multifaceted disease progression, however complex, allows circulating miRNAs to be involved in all steps of atherosclerosis (Figure 2). The interaction of miRNA with target mRNAs, especially, and timing of action in high-risk populations, is dynamic and highly dependent on the affinity of miRNA interaction, subcellular location of miRNAs, the abundance of miRNAs and target mRNAs, and the affinity of miRNA-mRNA interactions [26].

### 2.1. Endothelial Cells Regulation and Inflammatory Response

Endothelial cells are crucial in maintenance of vascular integrity and homeostasis [29]. Any alteration in the regulation of endothelial cells may provoke plaque build-up and contribute to the early stage of atherosclerosis and is reported to contribute towards vasospastic angina [25]. Patients with ischemia and non-obstructive coronary artery disease (INOCA), microvascular angina and vasospastic angina are frequently linked to microvascular dysfunction [30,31]. Under unfavoured conditions such as oxidative stress, a programmed cell death mechanism called apoptosis will be triggered to remove damaged cells and maintain tissue homeostasis. Shi and Chen [32] demonstrated the regulatory effects of miR-370 in coronary atherosclerosis by targeting the forkhead box protein O1 (*FOXO1*) gene. The *FOXO1* helps regulate endothelial cell apoptosis and cellular homeostasis when oxidative stress or redox signalling is present. Up-regulation of miR-370 in patients with coronary atherosclerosis hindered cellular apoptosis by reducing the expression of the *FOXO1* gene and enhancing endothelial cell migration and invasion.

Another study by Wu and colleagues [33] revealed the function of miR-145 in regulating vascular endothelial cells and inflammation response by targeting the same *FOXO1* gene. The miR-145 expression was found to be low in patients with ACS and negatively correlated with endothelial injury biomarkers and pro-inflammatory cytokines. The apoptotic cells released from the endothelial activation can transport miRNAs to atherosclerotic lesions and this uptake may explain the reduction of circulating miRNAs in patients [34]. Followed by a subsequent in vivo study, the overexpression of miR-145 reduced the concentration of endothelial injury markers and inflammatory cytokines, leading to the promotion of cell proliferation and migration, thus suggesting the protective role of miR-145 in the inflammatory response of ACS.

The talin-1 (*TLN1*) gene is the essential constituent of the extracellular matrix (ECM) and it plays a role in the maintenance of tissue integrity [35]. Both miR-182-5p and miR-9-5p were highly expressed in patients with coronary artery disease (CAD) and they both downregulated the TLN1 gene. The significant reduction in *TLN1* expression affected the ECM constituents and may contribute to the early stage of CAD by promoting the release of inflammatory mediators to trigger an inflammatory response.

Another miRNA involved in the regulation of endothelial cells is miR-451b, which targets and downregulates the vascular endothelial growth factor A (*VEGF-A*) gene [36]. The *VEGF-A* serves a role in stimulating the release of anti-apoptotic proteins and nitric oxide synthesis, which is crucial in the inflammatory response [37]. Upregulation of miR-451b decreases the expression of VEGF-A, leading to inhibition of cell proliferation and increased cell apoptosis.

Under normal circumstances, cell adhesion molecules of endothelial cells will not be activated to mediate the migration of leukocytes [38]. However, oxidised low-density lipoprotein (OX-LDL) will stimulate the endothelial inflammatory response and infiltration of leukocytes. Analysis of plasma samples showed that miR-381 was downregulated in patients with IHD and expression of the C-X-C chemokine receptor type 4 (*CXCR4*) target gene was increased, suggesting their inverse correlation. High expression of *CXCR4* in IHD was proved to protect endothelial cells against inflammatory damage with the stimulant of OX-LDL. With the inhibition of miR-381, cell proliferation activity was reduced, and higher expression of inflammatory cytokines was released, thus, promoting early cellular apoptosis.

Recent research suggests that co-morbidities-related microvascular dysfunction (MVD) and systemic low-grade inflammation are significant factors in the development of heart failure with preserved ejection fraction (HFpEF). The HFpEF is intricate and diverse, contributing to 22% to 73% of heart failures, and is reported to impact the availability of nitric oxide leading towards endothelial dysfunction [39].

### 2.2. Activation of Monocytes and Differentiation of Macrophages

Activated endothelial cells will release proinflammatory cytokines and adhesion molecules to provoke the differentiation of monocytes [40]. Upon stimulation by excessive lipids, macrophages will engulf necrotic cells and lipids to become foam cells and trigger more inflammatory responses that only aggravate the condition [22]. Zhang and colleagues (2018) demonstrated the role of miR-155 in the activation of macrophages by targeting cholesterol ester hydrolase (CEH), class A scavenger receptor (SR-A), and ATP binding cassette transporter A1 (ABCA1) proteins [22]. Usually, miR-155 is involved in the body’s immune system by regulating the secretion of inflammatory factors in T cells, B cells, monocytes, and macrophages. Overexpression of miR-155 significantly increased the level of CEH and ABCA1 proteins, resulting in increased lipid efflux in macrophages, reduced intracellular lipid accumulation, and inhibited the formation of foam cells. In contrast, overexpression of miR-155 inhibited the expression of SR-A that recognizes the specific binding sites of OX-LDL on macrophages and promotes cholesterol accumulation leading to foam cell formation.

Yang and colleagues [41] showed the role of miR-23a-5p in promoting plaque instability and IHD development by influencing the expression of *ABCA1* and ATP binding cassette subfamily G member 1 (*ABCG1*). The OX-LDL can activate miR-23a-5p and repress the expression of *ABCA1* and *ABCG1* that regulates reverse cholesterol transport. The reverse cholesterol transport mechanism is crucial for macrophages to release excessive cholesterol that helps to reduce inflammation and prevent apoptosis. Both *ABCA1* and *ABCG1* transporters have complementary functions in which *ABCA1* mediates the removal of cholesterol to lipid-free apolipoproteins while *ABCG1* transports cholesterol to HDL. In IHD, downregulation of *ABCA1* and *ABCG1* will inhibit the efflux of cholesterol and promote foam cell formation by allowing macrophages to take up excessive cholesterol uncontrollably.

Similar findings were seen by Lu and colleagues [42], whereby miR-320b was highly expressed in CAD patients, and it increased the formation of macrophages-derived foam cells under the stimulation of OX-LDL. This finding suggested that miR-320b plays a role in the regulation of cholesterol efflux and the pathogenesis of atherosclerosis. The miR-320b directly targeted ABCG1 and endonuclease-exonuclease-phosphatase family domain containing 1 (*EEPD1*). Meanwhile, *EEPD1* regulated the expression of *ABCA1* by controlling its activity in mediating cholesterol efflux. Further analysis on a miR-320b treated mice model revealed the effects of a reduced cholesterol efflux rate in macrophages, impaired lipid profile, increased inflammatory response, and promoted atherosclerotic plaque formation.

### 2.3. Plaque Angiogenesis

Inflammatory factors such as cytokines and chemokines released by inflammatory plaque can trigger angiogenesis [43]. Cytokines, notably the tumour necrosis factor-alpha (TNF-α), when lowly expressed can induce inflammation and promote angiogenesis through activation of tumour necrosis factor receptor 2 (*TNFR2*). The miRNA involved in plaque angiogenesis is miR-342-5p, which targets the WNT family member 3A (*WNT3A*) [44]. The upregulation of miR-342-5p decreased the expression of its targeted *WNT3A* gene in the *Apoe*^−/−^ mice model. Downregulation of miR-342-5p causes a reduction in micro vessel density, which suggests its inhibition role in preventing plaque angiogenesis in IHD.

The miR-21 was associated with plaque angiogenesis by targeting the *PTEN* [45]. The presence of Angiotensin II (*ANGII*) upregulated miR-21 in the endothelial cells by activating the signal transducer and activator of transcription 3 (*STAT3*). The highly expressed miR-21 inhibited the expression of phosphatase and the tensin homolog (*PTEN*), increased cell proliferation, migration, and capillary tube formation that ultimately increased the formation of new blood vessels. Inhibiting the miR-21 expression, or altering the STAT3 pathway, can therefore decrease the plaque angiogenesis induced by *ANGII*.

### 2.4. VSMC Proliferation and Differentiation

Intending to determine novel miRNA targets associated with VSMC proliferation and differentiation, Woo and colleagues [46] conducted research to evaluate the miRNA profiles of patients with MI. Their study revealed thirteen differentially expressed miRNAs clusters and four differentially expressed genes through network analysis. Muscle blind-like splicing regulator 1 (MBNL1), a novel gene that has not been reported with its role in human VSMC differentiation, was selected to undergo further analysis. Overexpression of hsa-miR-30b-5p decreased the expression of MBNL1 in the human VSMC culture experiment and reduced the level of common markers of VSMC differentiation.

Another study performed by Lai and colleagues [47] revealed the function of miR-574-5p in VSMC proliferation by targeting zinc finger DHHC-type palmitoyltransferase 14 (ZDHHC14). The miR-574-5p was upregulated in patients with coronary artery disease compared to the healthy controls. This overexpressed miR-574-5p reduced the expression of ZDHHC14 protein and contributed to IHD development by promoting VSMC proliferation and inhibiting cell apoptosis. Downregulating miR-574-5p serves the opposite function and therefore may be used as diagnostic biomarkers and therapeutic targets in IHD.

### 2.5. Fibrous Cap Destabilisation and Plaque Rupture

Vulnerable plaques that are likely to rupture consist mostly of high inflammatory cells with a large necrotic core protected by only a thin fibrous cap [48]. The stability of plaque in coronary arteries largely depends on its structure rather than its size [49]. The experiment conducted by He and colleagues (2019) demonstrated that miR-21 inhibited the expression of *PTEN* and promoted the expression of the matrix metalloprotease-2 (*MMP-2*) gene that destroys the integrity of the fibrous cap, leading to rupture of plaque. The overexpression of miR-21 results in reduced fibrous cap thickness, increased necrotic core area, and increased the number of macrophages around the fibrous cap, thus aggravating plaque instability.

Chen and colleagues [50] analysed the expression and role of miR-124-3p in collagen synthesis by using *Apoe*^−/−^ mice. The study revealed that miR-124-3p was highly expressed in VSMCs and it plays a role in atherosclerotic plaque stability. The miR-124-3p inhibited the expression of type I and III collagen, which was mainly synthesised by smooth muscle cells and reduced the number of VSMCs by targeting prolyl 4-hydroxylase subunit alpha 1 (*P4HA1*). Taken together, miR-124-3p ultimately increased the plaque vulnerability and contributed to fibrous cap thinning by influencing the function of VSMCs. Table 1 summarises miRNA appraised in this review.

## 3. Therapeutic Potential of miRNAs in IHD

Although the mechanism of action is different from traditional therapy, miRNA modulators might provide a more effective and long-lasting remedy with its bioavailability advantage that has been proven efficient in in-vivo studies [51]. Targeting miRNAs with anti-miRNAs, which are modified antisense oligonucleotides carrying the complementary reverse sequence of a mature miRNA, can neutralise the function of aberrantly expressed miRNAs and promote desired gene expression, whereas the use of miRNA mimics can stimulate the natural role of miRNAs to suppress gene expression and bring beneficial effects. Combination therapy of miRNAs with physical exercise has shown promising results in regression of coronary plaque burden [52]. However, the delivery of an appropriate therapeutic range of miRNAs to arteries is tricky considering the low effectiveness of direct injection [53]. Systemic administration of miRNA therapeutics poses a risk of degradation by phagocytic immune cells such as macrophages and monocytes that help to remove complex RNAs from the body [54]. Thus, the discovery of engineered miRNA carriers may potentially solve these bottlenecks by promoting cellular uptake and reducing extracellular degradation. Recently, the nonviral miRNA delivery methods using liposome nanoparticles have gained emerging attention with their success in delivering miR-153-3p for treating MI [55]. Another delivery method using cell-free therapy involving stem cell-derived exosomes provides high stability and minimal side effects with low immunogenicity [56]. The need for engineered delivery strategies is crucial for miRNA-based therapy with the hope to reduce the possibility of off-targeting transcripts expression that may provoke any safety issues. Table 2 summarises recent studies on therapeutic delivery of miRNA.

## 4. Caveats of Using miRNA as Diagnostic Biomarker in IHD

Although the knowledge of the role of miRNAs in the pathogenesis of IHD has improved over the decades, there is still a lack of existing research focusing on identifying and validating miRNAs as IHD diagnostic markers in clinical practice [65]. The challenges faced are their complex detection method and may incur an economic burden on society. Standard protocol on optimization is in demand to simplify the work of laborious miRNA isolation and its expression levels estimation [13]. Another significant drawback focuses on disease specificity due to the properties of miRNAs that are expressed differently in various diseased states and tissues [9]. Other confounding factors such as population differences, gender and IHC comorbidities should be taken into consideration, as it is reported to affect the profiles of circulating miRNA [66]. Furthermore, miRNAs are not tissue or cell type specific and thus the aberrant expression of particular miRNAs in different disease states may produce false signals and make it harder for precision diagnosis [67].

## 5. Conclusions

To date, many preclinical studies have shown various miRNAs that may potentially regulate different complex cellular processes and their involvement in IHD. However, this developing field still lacks in-depth information about the functional significance of miRNAs’ expression and their regulatory mechanism in IHD. Because a single miRNA can concurrently target multiple genes through different signalling pathways, manipulating the expression of a specific miRNA can affect the whole biological process and pose a risk of unknown pathological outcomes. Any miRNA-based interventions should be designed and planned carefully to make their significant usage as a theranostic target for IHD. The circulating miRNAs can significantly aid in early clinical diagnosis. They may provide a novel therapeutic approach in the near future by inventing personalised treatments targeted at specific stages of atherosclerosis to improve patient prognoses for long-term complications of IHD.

## Figures and Tables

**Table 1:** Role of miRNAs in ischemic heart disease.

Steps of Atherosclerosis	miRNA	Regulation	Model	Target Genes	Specific Roles of miRNA	Reference
Endothelial cells regulation and inflammatory response	miR-370	up	Human peripheral blood mononuclear cells Cell line: HUVEC	*FOXO1*	- Promotes endothelial cell migration and invasion - Inhibits apoptosis	[32]
	miR-145	down	Human serum Sprague –Dawley mice	*FOXO1*	Increases the concentration of endothelial injury biomarkers and the inflammatory cytokines	[33]
	miR-182-5pmiR-9-5p	up	Human serum Cell line: HEK293T	*TLN1*	- Interferes the ECM constituents	[35]
	miR-451b	up	Human peripheral blood Cell line: HUVEC	*VEGF-A*	- Inhibits cell proliferation - Promotes cell apoptosis	[36]
	miR-381	down	Human plasma Cell line: HUVEC	*CXCR4*	- Promotes release of inflammatory cytokines - Reduces cell proliferation - Promotes early apoptosis	[38]
Activation of monocyte and differentiation of macrophage	miR-155	up	Human THP-1 cells	*CEH* *ABCA1* *SR-A*	- Increases cholesterol efflux in macrophages - Decreases intracellular lipid accumulation - Inhibits foam cells formation	[22]
	miR-23a-5p	up	Human peripheral blood plasma *Apoe*^−/−^ mice Raw264.7 cells and HEK293T cells	*ABCA1* *ABCG1*	- Decreases cholesterol efflux in macrophages - Promotes foam cells formation	[41]
	miR-320b	up	Human peripheral blood mononuclear cells *Apoe*^−/−^ mice Human THP-1 cells, Raw264.7 cells, HEK293T, HUVEC	*ABCG1* *EEPD1*	- Reduces cholesterol efflux rate in macrophages - Promotes inflammatory response - Impairs lipid profile - Promotes atherosclerotic plaque formation	[13]
Plaque angiogenesis	miR-342-5p	up	*Apoe*^−/−^ mice and C57BL/6J mice	*WNT3A*	- Interferes the micro vessel density level	[44]
	miR-21	N/A	Cell line: HMECs	*PTEN*	- Increases endothelial cell proliferation and migration - Increases angiogenesis	[45]
VSMC proliferation and differentiation	hsa-miR-30b-5p	up	Human aortic wall tissue VSMC cell lines	*MBNL1*	Reduces the common markers level for VSMC differentiation	[46]
	miR-574-5p	up	Human serum Cell culture	*ZDHHC14*	- Promotes VSMC proliferation - Inhibits cellular apoptosis	[47]
Fibrous cap destabilisation and plaque rupture	miR-21	N/A	Human blood samples	*PTEN* *MMP-2*	- Reduces fibrous cap thickness - Increases necrotic core area - Increases the macrophages surrounding fibrous cap - Promotes plaque instability	[49]
	miR-124-3p	up	*Apoe*^−/−^ mice Human aortic smooth muscle cell lines	*P4HA1*	- Inhibits collagen synthesis of VSMCs - Reduces plaque stability	[45]

**Table 2:** Therapeutic delivery of miRNA as biomarker for IHD.

Model	Intervention	Outcomes	Reference
HL-1 cell line transfected with microRNA	administration of miR-22 anti-miRs	activates cardiac autophagy to prevent post-infarction remodeling and improve cardiac function	[57]
Murine model	Lentivirus-mediated miR-99a delivery	improved survival rate and cardiac function	[58]
Rat model	adenovirus -delivered miR-214 or miR-21 improved	improved LV remodeling and decreased myocardial apoptosis	[59,60]
Rat model	administration of miR-320 anti-miRs	reduced the degree of myocardial fibrosis and apoptosis in LV remodeling	[61]
Rat model	inhibition of miR-132	rescues cardiac hypertrophy and heart failure	[62]
Mouse model	administration of a locked nucleic acid anti-miR-652	attenuation of cardiac hypertrophy. Improved heart function was associated with reduced cardiac fibrosis	[62]
Porcine model	single intracoronary administration of encapsulated anti-miR-92a	prevented left-ventricular remodeling	[63]
Porcine model	administration of locked nucleic acid modified anti-miR-15	cardiomyocytes showed resistant to hypoxia-induced cardiomyocyte cell death	[64]

## Data Availability

Not applicable.
